# Correlation analysis of endoscopic manifestations and eradication effect of *Helicobacter pylori*

**DOI:** 10.3389/fmed.2023.1259728

**Published:** 2023-08-29

**Authors:** Xin Luo, Hui Li, Li He

**Affiliations:** ^1^Hepatic Surgery Center, Tongji Hospital, Tongji Medical College, Huazhong University of Science and Technology, Wuhan, Hubei, China; ^2^Department of Geriatrics, Tongji Hospital, Tongji Medical College, Huazhong University of Science and Technology, Wuhan, Hubei, China; ^3^Department of Gastroenterology, Tongji Hospital, Tongji Medical College, Huazhong University of Science and Technology, Wuhan, Hubei, China

**Keywords:** *Helicobacter pylori*, *H. pylori* eradication, *H. pylori* resistance, endoscopic manifestation, personalized treatment

## Abstract

**Background:**

*Helicobacter pylori* (*H. pylori*) infection is the most important risk factor for gastric cancer. Eradication of *H. pylori* significantly reduces the incidence and mortality of gastric cancer. *H. pylori* resistance to antibiotics and a gradual decline in eradication rates are gaining more and more attention. Our study aimed to address the correlation between endoscopic manifestations and the eradication effect of *H. pylori*.

**Methods:**

We retrospectively reviewed outpatients in our hospital with *H. pylori* infection undergoing eradication therapy from January 2022 to March 2023. Both the primary diagnosis and eradication of *H. pylori* after treatment were confirmed by a ^13^C urea breath test. Patients were treated with a proton pump inhibitor (PPI)-based quadruple therapy. Clinical characteristics and endoscopy manifestations within 7 days before or after patients were diagnosed with *H. pylori* infection were analyzed.

**Results:**

From January 2022 to March 2023, a total of 323 patients were enrolled in this study. There were 138 male patients and 185 female patients. The mean age of patients was 45.62 ± 13.04 years. The *H. pylori* initial eradication rate was 82.0%. Univariate analysis of factors affecting *H. pylori* eradication showed that sex, age, and endoscopic manifestations including diffuse redness, multiple white, and flat elevated lesions, and atrophy were significantly associated with the failure of *H. pylori* eradication therapy. A multivariable logistic regression model analysis of these five factors showed that patients aged over 60 years with multiple white and flat elevated lesions in the endoscopic examination are significantly less likely to eradicate *H. pylori* with empirical quadruple therapy. On the other hand, patients with diffuse redness were significantly more likely to eradicate *H. pylori* infection with empirical quadruple therapy.

**Conclusion:**

Our study shows that age over 60 years old, multiple white and flat elevated lesions in endoscopic examination are independent risk factors of initial *H. pylori* eradication failure with empirical quadruple therapy, while diffuse redness in endoscopic examination is a protective factor of initial *H. pylori* eradication failure with empirical quadruple therapy, while diffuse redness in endoscopic examination is a protective factor. For patients with these risk factors, a drug sensitivity test or *H. pylori* resistance gene mutation detection may be more appropriate. However, further mechanism studies or prospective studies are needed to prove our findings.

## 1. Introduction

*Helicobacter pylori* (*H. pylori*) is a gram-negative microaerophilic bacterium that infects the epithelial lining of the stomach. It is one of the most prevalent bacterial infections in human beings worldwide. It is estimated that 50% of the global population is infected with *H. pylori* (1). Numerous researchers have discovered that *H. pylori* infection is directly associated with gastric diseases, including chronic gastritis, gastric and duodenal peptic ulcer, gastric adenocarcinoma, and gastric mucosa-associated lymphoid tissue (MALT) lymphoma (2, 3). In 1994, the International Agency for Research on Cancer classified *H. pylori* as a class I carcinogen for gastric cancer (4). Meanwhile, several large cohort studies have highlighted the importance of *H. pylori* eradication and shown that the eradication of *H. pylori* significantly decreases gastric cancer incidence with no increase in the likelihood of adverse consequences (5, 6). Thus, experts have reached a consensus that the screening and eradication of *H. pylori* should be adopted as a public health policy to prevent gastric cancer in high-risk populations.

Bismuth quadruple therapy (BQT) is the main empirical therapy for initial *H. pylori* eradication therapy in most areas. It consists of a proton pump inhibitor (PPI) or histamine-2 receptor antagonist, bismuth, and two antibiotics. Amoxicillin, clarithromycin, levofloxacin, metronidazole, furazolidone, and tetracycline are optional antibiotics. The course of treatment is usually 10 or 14 days (7–9). However, the eradication rate ranges from 77.6% to 98.0% (7, 10). Large-scale *H. pylori* eradication treatment and inappropriate treatment regimens have led to increasing resistance rates to multiple antibiotics, which results in a gradual decline in *H. pylori* eradication rates, especially in areas with high *H. pylori* prevalence. Currently, repeated empirical eradication of *H. pylori* treatment is common in clinical practice, which will result in a great waste of medical resources and an increased psychological burden on patients and their relatives. Therefore, successful eradication of *H. pylori* on initial treatment is very important. Nowadays, drug sensitivity tests and *H. pylori* resistance gene mutation detection can enable personalized, promising salvage treatments and achieve comparably high eradication rates (11). However, the drug sensitivity test for *H. pylori* takes a long time and is hampered by a relatively high rate of false negatives (11). Meanwhile, *H. pylori* resistance gene mutation detection is not easy to get access, especially in developing countries where the *H. pylori* infection rate is relatively higher (12). Thus, further study in distinguishing high-risk patients is needed to better customize the application of *H. pylori* resistance gene mutation detection and decrease the failure of *H. pylori* eradication. As *H. pylori* infection is directly associated with gastric diseases, which usually cause stomach upset, patients infected with *H. pylori* often underwent endoscopy examinations. Is there a correlation between the endoscopic manifestations and *H. pylori* eradication failure? Is it possible that we distinguish patients who have a high risk of failing the empirical BQT based on their endoscopic manifestations? To address these questions, we conducted this retrospective study. In this study, we described the clinical characteristics and endoscopic manifestations of the enrolled outpatients in our hospital with *H. pylori* infection undergoing eradication therapy. We also evaluate the risk factors for *H. pylori* eradication failure, especially the correlation between endoscopic manifestations and *H. pylori* eradication failure.

## 2. Methods

### 2.1. Patients

From January 2022 to March 2023, 323 outpatients at our hospital were enrolled. The following were the inclusion criteria: 1. Patients aged at least 18; 2. Patients underwent endoscopy examination within 7 days of being diagnosed with *H. pylori* infection; 3. After endoscopy examination, patients were treated with a PPI-based quadruple therapy: PPI (omeprazole 40 mg or pantoprazole 40 mg or rabeprazole 10 mg or esomeprazole 20 mg); clarithromycin (500 mg); amoxicillin (1,000 mg), and bismuth potassium citrate capsules (220 mg) each twice/day for 14 days; 4. Patients had *H. pylori* tests 1–3 months after the quadruple *H. pylori* eradication therapy; 5. Both primary diagnosis and eradication of *H. pylori* after therapy were confirmed by the ^13^C urea breath test (^13^C-UBT), which was recommended by both Chinese and American guidelines (7, 13). Before undergoing 13C-UBT, patients were required to stop taking antibiotics, bismuth, proton pump inhibitors, H2-receptor antagonists, or other medicines that might interfere with the result of the examination for at least 4 weeks. Clinical characteristics, including patients' age, sex, if they have clinical symptoms such as epigastric pain, nausea, or dyspepsia, and the duration of their symptoms were obtained from their medical records. We retrospectively analyzed these data. This study was approved by the Ethics Committee of Tongji Medical College, and all patient information was kept private.

### 2.2. Methodology of ^13^C-UBT

Each subject was requested to swallow a pill containing 75 mg ^13^C urea with 20 ml of water in the morning. Exhaled air was collected in sampling tubes 30 min later. ^13^CO_2_ values were determined using a gas isotope ratio mass spectrometer, and a delta over baseline (DOB) value was used to express the difference between 30 min and baseline. The concentration of ^13^CO_2_ at 30 min that exceeded the baseline by more than 3.8 parts per thousand (>0.38%) was regarded as a positive indicator of *H. pylori* infection.

### 2.3. Endoscopic manifestations

We selected the endoscopic manifestations of the enrolled patients as potential factors that might affect the eradication of *H. pylori* infection. These endoscopic manifestations included: reflux esophagitis, gastritis, peptic ulcers, and duodenitis. Because the Kyoto classification is a kind of gastritis classification that is associated with *H. pylori* infection (14), for the manifestation of gastritis, we selected diffuse redness, raised erosion, depressive erosion, multiple white and flat elevated lesions, and atrophy from the Kyoto classification.

Data were analyzed using SPSS statistical package version 27.0. Descriptive statistics such as percentages, means, and standard deviations were used to describe the data. The chi-square test was used to assess failure-success differences in *H. pylori* eradication. Multivariate analyses were performed using binary non-conditional logistic regression to identify predictors of failure of eradication using BQT. The Hosmer–Lemeshow test was checked to assess the model's fitness to conduct logistic regression. The odd's ratio with 95% confidence intervals was calculated for each of the independent variables using a P-value of < 0.05 as the level of significance.

## 3. Results

### 3.1. Characteristics of patients

From January 2022 to March 2023, a total of 323 patients were enrolled in this study. Their clinical characteristics are listed in Table 1. There were 138 male patients and 185 female patients. The male-to-female ratio was 1:1.34. The mean age of patients was 45.62 ± 13.04 years. The youngest patient was 18 years old, whereas the oldest was 75 years old. Most people were between 40 and 60 years old. The overall *H. pylori* initial eradication rate (success of therapy) was 82.0%, while 58 patients (18%) failed to eradicate *H. pylori* with the empirical BQT. A total of 297 (92.0%) patients had symptoms when they came to the clinic, while 26 (8.0%) patients had no symptoms. A total of 168 (56.6%) patients had clinical symptoms for no more than 6 months. A total of 129 (43.4%) patients' symptoms lasted for more than 6 months. In addition, the number and percentage of patients for each kind of endoscopic manifestation are shown in Table 1. There were 101 (31.3%) patients with diffuse redness, 97 (30%) patients with raised erosion, 50 (15.5%) patients with depressive erosion, 18 (5.6%) patients with multiple white and flat elevated lesions, 164 (50.8%) patients with atrophy, 30 (9.3%) patients with reflux esophagitis, 67 (20.7%) patients with peptic ulcer, and 54 (12.4%) patients with duodenitis.

**Table 1:** Clinical characteristics and endoscopic manifestations of 323 patients.

**Variables**	**Sample-size, *n* (%)**
**C13 test after HP eradication therapy**	
Negative	265 (82.0)
Positive	58 (18.0)
**Sex**	
Male	138 (42.7)
Female	185 (57.3)
**Age**	
≤ 20	7 (2.2)
21–40	101 (31.3)
41–60	176 (54.4)
>60	39 (12.1)
**Presence of clinical symptoms**	
Yes	297 (92.0)
No	26 (8.0)
**Duration of clinical symptoms**	
≤ 6month	168 (56.6)
> 6months	129 (43.4)
**Diffuse redness**	
Yes	101 (31.3)
No	222 (68.7)
**Raised erosion**	
Yes	97 (30.0)
No	226 (70.0)
**Depressive erosion**	
Yes	50 (15.5)
No	273 (84.5)
**Multiple white and flat elevated lesions**	
Yes	18 (5.6)
No	305 (94.4)
**Atrophy**	
Yes	164 (50.8)
No	159 (49.2)
**Reflux esophagitis**	
Yes	30 (9.3)
No	293 (90.7)
**Peptic ulcer**	
Yes	67 (20.7)
No	258 (79.3)
**Duodenitis**	
Yes	54 (12.4)
No	269 (87.6)

### 3.2. Endoscopic manifestation and *H. pylori* eradication rate of patients

**Figure 1:**
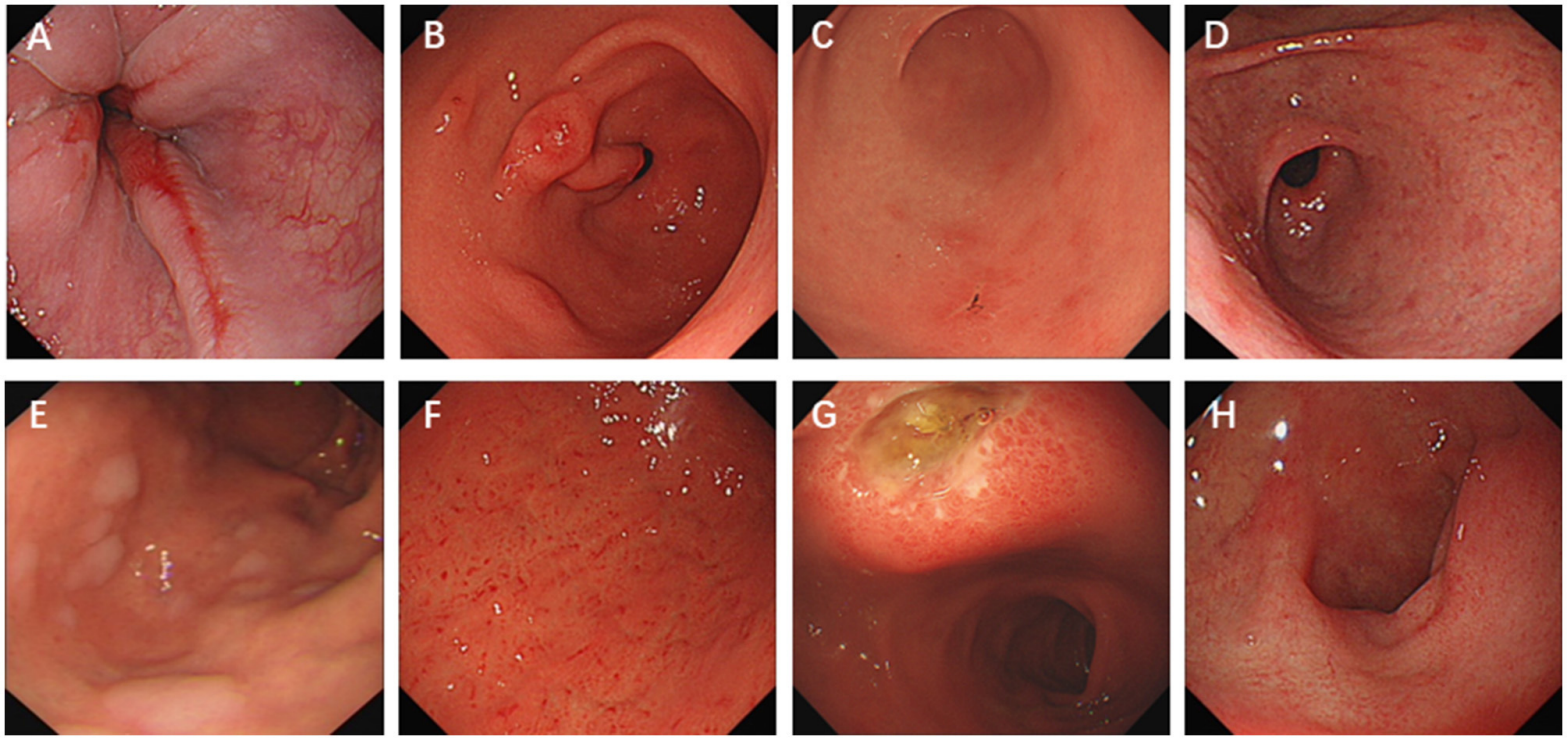
Typical images of endoscopic manifestations selected in this study. **(A)** Reflux esophagitis, gastritis, **(B)** raised erosion, **(C)** depressive erosion, **(D)** atrophy, **(E)** multiple white and flat elevated lesions, **(F)** diffuse redness, **(G)** peptic ulcer, and **(H)** duodenitis.

Typical endoscopic manifestation pictures are shown in Figure 1. Univariate analysis of factors that might affect *H. pylori* eradication failure showed that sex, age, and endoscopic manifestations including diffuse redness, multiple white and flat elevated lesions, and atrophy were significantly associated with the failure of *H. pylori* eradication therapy (Table 2).

**Table 2:** Univariate analysis of the factors affecting *H. pylori* eradication failure.

**Variables**	**Variable categories**	**C13 test after HP eradication therapy**		***P***
		**Negative**, ***n*** **(%)**	**Positive**, ***n*** **(%)**	
Sex	Male	120 (87.0)	18 (13.0)	0.047
	Female	145 (78.4)	40 (21.6)	
Age	≤ 60	242 (85.2)	42 (14.8)	<0.001
	>60	23 (58.9)	16 (41.1)	
Presence of clinical symptoms	Yes	245 (82.5)	52 (17.5)	0.478
	No	20 (76.9)	6 (23.1)	
Duration of clinical symptoms	≤ 6 month	140 (83.3)	28 (16.7)	0.156
	> 6 months	99 (76.7)	30 (23.3)	
Diffuse redness	Yes	92 (91.1)	10 (8.9)	0.010
	No	173 (77.9)	48 (22.1)	
Raised erosion	Yes	78 (80.4)	19 (19.6)	0.617
	No	187 (82.7)	39 (17.3)	
Depressive erosion	Yes	39 (78.0)	11 (22.0)	0.418
	No	226 (82.8)	47 (17.2)	
Multiple white and flat elevated lesions	Yes	9 (50.0)	9 (50.0)	<0.001
	No	256 (83.9)	49 (16.1)	
Atrophy	Yes	124 (75.6)	40 (24.4)	0.002
	No	141 (88.7)	18 (11.3)	
Reflux esophagitis	Yes	26 (86.7)	5 (16.7)	0.780
	No	239 (81.6)	53 (18.4)	
Peptic ulcer	Yes	56 (83.6)	11 (16.4)	0.629
	No	209 (81.0)	49 (19.0)	
Duodenitis	Yes	41 (75.9)	13 (24.1)	0.199
	No	224 (83.3)	45 (16.7)	

The failure rates of male and female patients were 13.0% and 21.6%, respectively (*P* = 0.047). The failure rate of patients who were aged more than 60 was as high as 41.4%, while the rate was 14.8% for patients who were aged not more than 60 (*P* < 0.001). Patients whose endoscopic findings included diffuse redness showed a failure rate of 8.9%, while the remaining patients had a significantly higher failure rate of 22.1% (*P* = 0.010). Patients whose endoscopic findings included multiple white and flat elevated lesions showed a failure rate of 50%, while patients who did not have multiple white and flat elevated lesions showed a significantly lower failure rate of 16.1% (*P* < 0.001). In addition, patients who were diagnosed with atrophy showed a failure rate of 24.4% and the remaining patients had a significantly lower failure rate of 11.3% (*P* = 0.002).

### 3.3. Multivariable analysis of the factors affecting the success of *H. pylori* eradication

Logistic regression analysis was performed by the abovementioned five factors selected through univariate analysis. Results revealed that age, diffuse redness, and multiple white and flat elevated lesions were factors affecting the result of the eradication therapy. Patients aged more than 60 years were significantly less likely to eradicate *H. pylori* infection compared to patients aged no more than 60 years (OR: 0.348, 95% CI:0.155–0.784, *p* = 0.011). Those patients with multiple white and flat elevated lesions were significantly less likely to eradicate *H. pylori* infection with empirical quadruple therapy compared to those without multiple white and flat elevated lesions (*p* = 0.002). On the other hand, patients with diffuse redness were significantly more likely to eradicate *H. pylori* infections compared to those without diffuse redness (*p* = 0.049) (Table 3).

**Table 3:** Multivariable analysis of factors affecting *H. pylori* eradication success.

**Variables**	***P***	**OR**	**95%CI**	
			**The lower limit**	**The upper limit**
**Sex**	0.079			
Male		1		
Female		1.796	0.935	3.448
**Age**	0.011			
≤ 60		1		
>60		0.348	0.155	0.784
**Diffuse redness**	0.049			
Yes		1		
No		0.470	0.221	0.998
**Multiple white and flat elevated lesions**	0.002			
Yes		1		
No		5.394	1.873	15.532
**Atrophy**	0.098			
Yes		1		
No		1.780	0.899	3.524

## 4. Discussion

*H. pylori* eradication is very important in curing chronic gastritis and peptic ulcers. Population-based eradication of *H. pylori* is also effective in reducing the incidence of gastric adenocarcinoma and gastric MALT (7, 15). Because of the wide application of empirical BQT in the eradication therapy of *H. pylori* infection, together with factors such as coccoid transformation, host CYP2C19 gene polymorphisms, and inappropriate treatment regimens, a gradual decline in *H. pylori* eradication rates has been reported in previous studies (16, 17). Improving the eradication rate on initial treatment is critical to reduce antibiotic resistance (18). Worldwide, the initial eradication rate ranges from 77.6% to 98.0% (7, 10). The eradication rate in this study ranges from 50% to 91.1%. Patients aged more than 60 and patients with endoscopic examination showing multiple white and flat elevated lesions have lower eradication rates. While patients with endoscopic examination showing diffused redness showed the highest eradication rate (91.1%).

Our study showed that the initial eradication rate for patients over 60 years old is lower. Some studies showed that the elderly population had no significantly increased resistance to antibiotics commonly used to eradicate *H. pylori* such as amoxicillin and furazolidone, but their resistance to quinolones (levofloxacin) and clarithromycin had increased (13, 19). It may decrease the eradication rate among elderly people. In addition, elderly people in China usually have a lower level of education. It significantly impacts patients' adherence to a multi-drug regimen with frequent side effects, which will affect the result of the initial eradication rate. Meanwhile, the elderly population often has one or more conditional diseases at the time of treatment, so choosing the proper drugs to improve the initial eradication rate is important. Therefore, drug sensitivity test or *H. pylori* resistance gene mutation detection may be critical in elderly people.

Diffuse redness is a very important endoscopic manifestation in the Kyoto classification. It indicates a current infection of *H. pylori* and is related to active inflammation. Multiple white and flat elevated lesions are pathologically characterized by a hyperplasia of the foveolar epithelium, in which inflammatory cell infiltration is extremely low (20). Multiple white and flat elevated lesions are considered to be associated with long-term PPI use (21). In this study, these two endoscopic manifestations were associated with the initial eradication rate. Our study showed that patients with diffuse redness had a higher success eradication rate. While patients with multiple white and flat elevated lesions had a lower success eradication rate. The mechanism for this phenomenon is unknown. The possible mechanisms may relate to the characteristics of the bacteria. Unique characteristics of different *H. pylori* subtypes may affect its drug resistance as well as its ability to induce active inflammation. An example is when non-mucoid spiral *H. pylori* turned into mucoid-coccoid form, its antibiotic resistance changed at the same time (22). This study is the first study about the correlation between *H. pylori* eradication rate and endoscopic manifestations. More research is needed in future to explore their relations and the underlying mechanism.

There are some limitations to this study. The first limitation is that our study population is from a single medical center, and selection bias may exist. It may result in a lack of statistical reliability. A multicenter prospective study is needed to confirm the results we have found. The second limitation is that this is a retrospective analysis of clinical record data without mechanism experiments. Thus, lab experiments are also needed to reveal the mechanism underlying the phenomenon.

## 5. Conclusion

Our study shows that age over 60 years old, multiple white and flat elevated lesions in endoscopic examination are independent risk factors of initial *H. pylori* eradication failure with empirical quadruple therapy, while diffuse redness in endoscopic examination is a protective factor of initial *H. pylori* eradication failure with empirical quadruple therapy, while diffuse redness in the endoscopic examination is a protective factor. For patients with these risk factors, a drug sensitivity test or *H. pylori* resistance gene mutation detection may be more appropriate. However, further mechanism studies or prospective studies are needed to prove our findings.

## Data availability statement

The original contributions presented in the study are included in the article/supplementary material, further inquiries can be directed to the corresponding author.

## Ethics statement

The studies involving humans were approved by Ethics Committee of Tongji Medical College. The studies were conducted in accordance with the local legislation and institutional requirements. The Ethics Committee/Institutional Review Board waived the requirement of written informed consent for participation from the participants or the participants' legal guardians/next of kin because all the information of patients were kept private.

## Author contributions

XL: Formal analysis, Resources, Writing—original draft, Writing—review and editing. HL: Formal analysis, Resources, Writing—review and editing. LH: Conceptualization, Project administration, Resources, Supervision, Writing—review and editing, Funding acquisition.
